# Investigation of MDMA Inhibitory Effect on CytochromeP450 3A4 in Isolated Perfused Rat Liver Model Using Tramadol

**DOI:** 10.34172/apb.2021.061

**Published:** 2020-08-05

**Authors:** Behjat Sheikholeslami, Zahra Tootoonchi, Hoda Lavasani, Yalda Hosseinzadeh Ardakani, Mohammadreza Rouini

**Affiliations:** Biopharmaceutics and Pharmacokinetics Division, Department of Pharmaceutics, Faculty of Pharmacy, Tehran University of Medical Sciences, Tehran, Iran.

**Keywords:** Tramadol, Ecstasy, CYP3A4, Liver perfusion, Metabolism

## Abstract

***Purpose:*** MDMA (methylenedioxymethamphetamine) is a synthetic compound, which is a structurally derivative of amphetamine. Also, it acts like an amphetamine, structurally, and functionally. MDMA uses mechanism-based inhibition, to inhibit isoenzyme CYP2D6. It can also inhibit other isoenzymes contributing to its metabolism, including CYP3A4 which is the most important member of the cytochrome P450 superfamily. Since more than 50% of drugs are metabolized by CYP3A4, its inhibition may cause harmful and even lethal drug interactions. Tramadol, as an opioid-like analgesic, is mainly metabolized into O-desmethyl tramadol (M1), by CYP2D6 and undergoes N-demethylation to M2, by CYP2B6 and CYP3A4. Due to the significant potential of abusing tramadol, either alone or in combination with MDMA, the rate of its toxicity and side effects may increase following possible MDMA relevant enzyme inhibition.

***Methods:*** Different doses of MDMA (1-10 mg/kg) were intraperitoneally administered to Wistar male rats of both control and treatment groups. Then, after one hour, their isolated livers were perfused by perfusion buffer containing tramadol (1 µg/mL). Afterward, perfusate samples were collected. They were analyzed by HPLC to determine the concentrations of tramadol and its metabolites.

***Results:*** MDMA administration in treatment groups reduced M1 production. On the other hand, by following the treatment with different MDMA doses, the M2 metabolic ratio increased by 46 to 101%.

***Conclusion:*** it seems that the regular doses of MDMA cannot inhibit the CYP3A4 activity.

## Introduction

MDMA or ecstasy, which is known as a stimulant substance, is a synthetic illicit drug, similar to amphetamine’s structure and function.^1^ According to the report of the United Nations Office on Drugs and Crime (UNODC) in 2017, the global number of MDMA consumers was estimated at 21.3 million among the population of 15-64 years old.^2^ High consumption of ecstasy can be attributed to its positive psychological effects including euphoria, increased self-confidence, and energy, feeling close to others, distortions in time and perception, enhanced enjoyment from the experience of sensory contact, etc. These effects usually remain up to 3-6 hours following ecstasy consumption.^3-5^ In general, the adverse effects of ecstasy are divided into short-term and long-term effects. After MDMA consumption, short-term effects take up to a week, while the long-term effects remain even years after cutting ecstasy or during the individual’s lifetime.^5-7^

**Figure 1:**
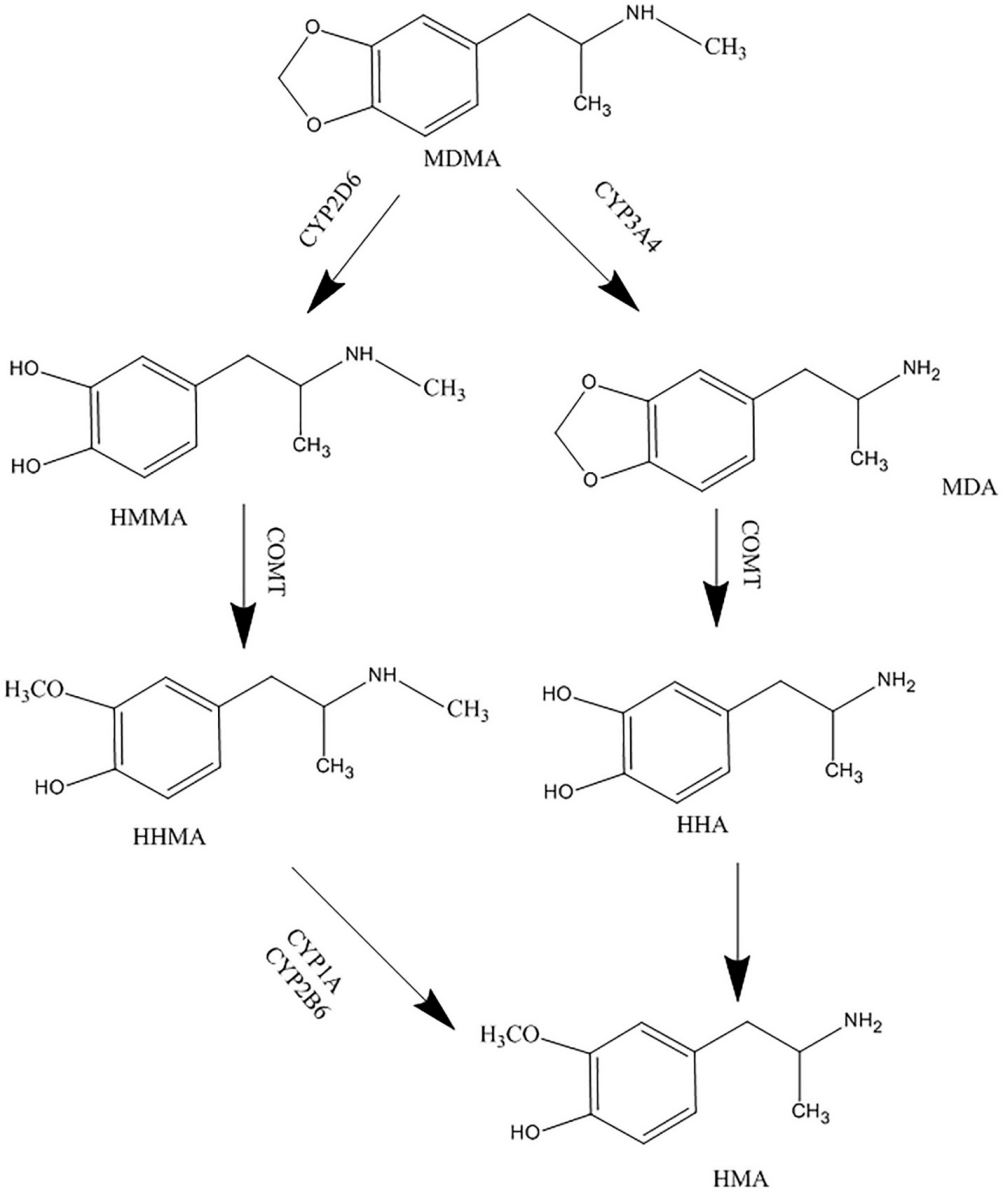
Metabolism pathway of MDMA.

Ecstasy is metabolized in the liver by different members of the cytochrome P450 family. MDMA is mainly O-demethylenated to 3, 4-dihydroxymethamphetamine (HHMA) by CYP2D6, and a lesser extent by CYP1A2 and CYP3A4. HHMA is then O-methylated to 4-hydroxy-3-methoxymethamphetamine (HMMA).^8-10^ N-demethylation is also catalyzed mainly by CYP3A4 and CYP2B6, which results in producing the active metabolite, 3, 4-methylendioxyamphetamine (MDA).^7,9^ The MDMA metabolic pathway is illustrated in Figure 1.

MDMA’s impact on hepatic cytochromes’ activities, is an important matter. In previous studies, it has been shown that, due to the presence of a methylenedioxy group in MDMA structure, it irreversibly inhibits CYP2D6.^11,12^ Throughout the metabolization of ecstasy into HHMA via CYP2D6, MDMA forms an ortho-quinone intermediate complex, causing a quasi-irreversible loss of enzyme function (known as mechanism-based inhibition).^13-15^ The intermediate complex may also have the same effect on the other enzymes, including CYP3A4, involving in MDMA metabolism.

**Figure 2:**
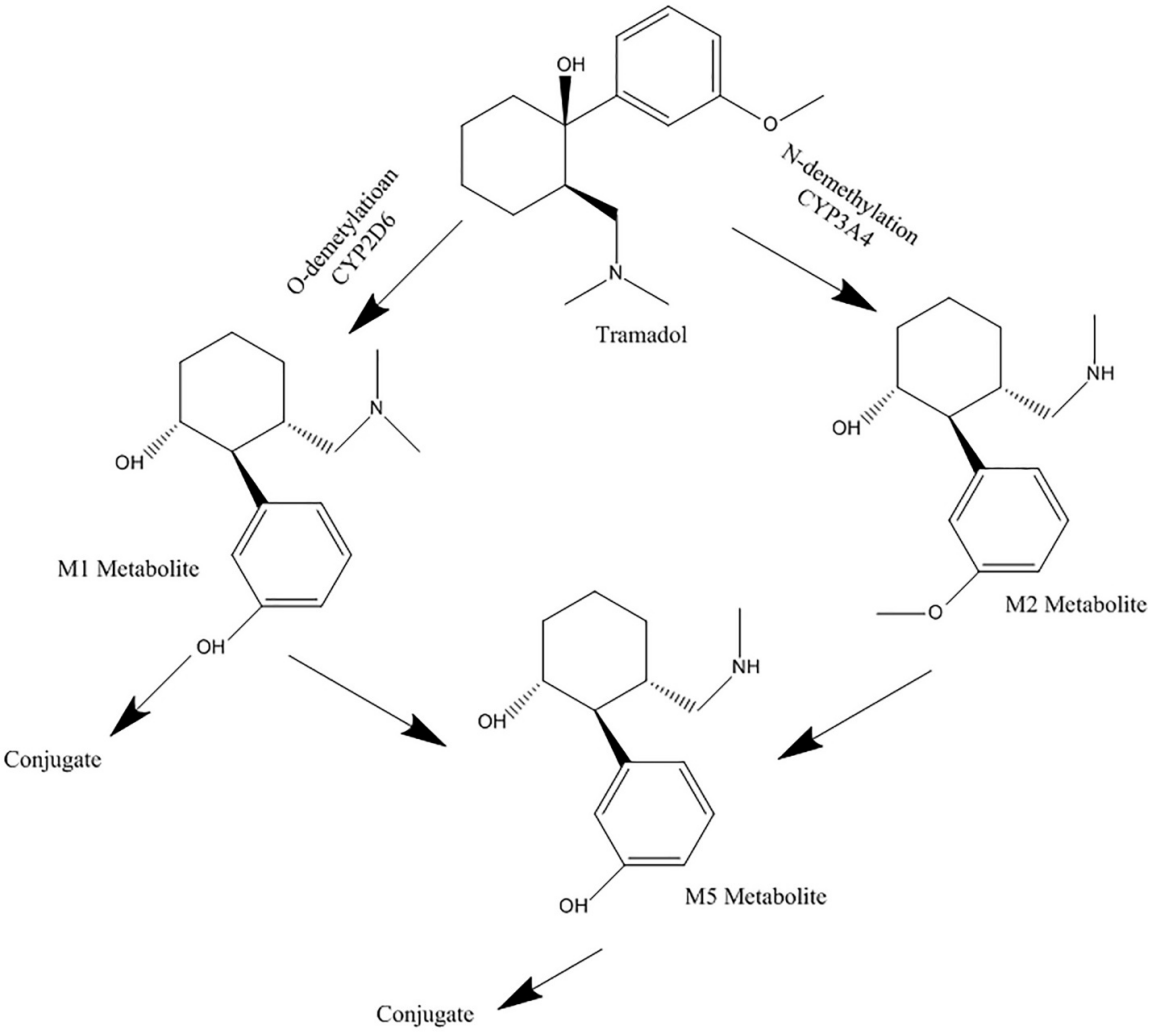
Metabolism pathway of tramadol

Tramadol is an analgesic opioid, which affects the body’s central system and is indicated to treat moderate to severe pain. The drug is usually available as a racemic mixture.^16^ Its analgesic effect is produced by two different mechanisms; binding to μ opioid receptors, and weak inhibition of norepinephrine and serotonin reuptake.^17,18^ Tramadol is mainly metabolized by CYP2D6 isoenzyme to O-desmethyl tramadol (M1), which is its active metabolite, in the liver. CYP3A4 and CYP2B6 catalyze the biotransformation of the parent drug, to N-desmethyl tramadol (M2).^18,19^ Both metabolites are then metabolized to N, O-didesmethyl tramadol (M5). It has been reported that CYP2D6 has an essential role in M5 production.^19^ Tramadol metabolic pathway is illustrated in Figure 2. According to the aforementioned effects of MDMA on CYP2D6 and CYP3A4 isoenzymes, and by considering the roles of these two in tramadol metabolism, and since the consumption rate of MDMA is significant among the youth, evaluating the possible MDMA and tramadol interaction, due to possible simultaneous use of ecstasy and tramadol, to predict the possible related toxicity seems necessary. Serotonin syndrome is one of the important predicted side effects of tramadol and MDMA interaction. Several studies have described the role of ecstasy as a CYP2D6 inhibitor and tramadol as a CYP2D6 probe.^11-15,20^ While there are a few numbers of specific studies about the effects of ecstasy on the function of CYP3A4.^21,22^ CYP3A4 is the most abundantly expressed P450 in the liver. It is responsible for the metabolism of more than 50% of common drugs^23,24^ including macrolide antibiotics, benzodiazepines, statins, etc.^25^ Therefore, further researches would be required to understand the inhibitory effects of MDMA on the CYP3A4 enzyme.

Isolated perfused rat liver model is a very common technique, for pharmacokinetic and drug interaction studies. In the mentioned technique, the vascular system and structure of the liver are preserved and isolated from the effects of other tissues and endogenous compounds.^26^ In the present study, the inhibitory effect of MDMA on CYP2D6 and CYP3A4 activities, contributing to tramadol metabolism, was investigated in an isolated perfused rat liver model.

## Materials and Methods

### Materials and animals

Pure powder of tramadol has been provided from Modava Company, and M1, M2, and M5 metabolites from Grünenthal (Aachen, Germany). Pure powder of ecstasy has been synthesized in the Pharmaceutical Chemistry laboratory, Faculty of Pharmacy, Tehran University of medical sciences. Also, paroxetine was used under the Paxil brand, 20 mg tablets. All chemical materials used in this study, have been provided by the Merck Company (Darmstadt, Germany). Millipore Direct-Q system (USA) was used for obtaining the ultrapure water consumed during the process of the study.

Adult male Wistar rats used in the present study weighed between 250-300 g. They were kept in the animal house of Faculty of Pharmacy, Tehran University of medical sciences, in a humidity-controlled animal care room at 25 ± 1°C temperature with a 12-hour light-dark cycle. They had access to chow and water ad libitum. Animals were divided into three groups, each containing four rats: control, treatment, and positive control.

### Preparation of drug solutions

Fifty milligrams of pure ecstasy powder was dissolved in 5 mL of normal saline. This solution (10 mg/mL) was considered as the stock solution. Other required concentrations (1 mg/mL and 5 mg/mL) were prepared from initial stock, diluted with normal saline.

Twenty-five milligrams of pure tramadol powder or its metabolites (M1, M2, and M5) was dissolved in 25 mL of methanol. The outcome, with a concentration of 1 mg/mL were considered as the stock solution. The pooled QC concentrations were prepared from the initial stocks by using the Krebs-Henseleit buffer.

Paroxetine with a concentration of 0.266 mg/mL, was used for this purpose. 20 mg tablet of paroxetine (Paxil) was completely crushed in a mortar. Then it was dissolved in 20 mL of deionized water. The resultant concentration, 1 mg/mL, was considered as the stock solution. To reach a concentration of 0.266 mg/mL of paroxetine, we transferred 6.65 mL of the stock solution into a 25 mL volumetric flask and then added deionized water to reach the final volume of 25 mL.

All of the solutions were kept in the refrigerator at 4°C temperature.

### Enzyme inhibition

One hour before the liver perfusion, the treatment groups were administered an intraperitoneal dose of ecstasy (1, 5, and 10 mg/kg), in three subgroups. The control group was just administered with the same volume of normal saline without ecstasy.^8^ In positive control (paroxetine group), rats were administered an oral paroxetine dose of 0.266 mg/kg, for three consecutive days. This was for comparing the inhibitory effects of MDMA and paroxetine, as a potent CYP2D6 inhibitor, on CYP2D6 activity. Animals were then anaesthetized at day four for liver perfusion.^27^

### Drug administration, liver perfusion, and sampling

All groups were anaesthetized by intraperitoneal injection of ketamine/xylazine (75/15 mg/kg). Their portal vein and inferior vena cava were cannulated with intravenous catheters 16–18 gauge. In order to perform liver perfusion, freshly prepared Krebs-Henseleit buffer (118 mM NaCl, 4.5 mM KCl, 2.75 mM CaCl_2_, 1.19 mM KH_2_PO_4_, 1.18 mM MgSO_4_, 25 mM NaHCO_3_ and 0.1% w/v glucose, equilibrated with 95% O_2_ and 5% CO_2_, pH 7.4 in 1 L of deionized water) was used as the perfusion buffer.^19^ Firstly, Krebs buffer was perfused for 5 min as a washing step. Then tramadol was added to the buffer to make the concentration of 1000 ng/mL. The subsequent buffer containing tramadol (1 µg/mL) was inserted into the liver via the portal vein and removed via inferior vena cava with a constant flow rate of 8.3 mL/min, and by using a peristaltic pump. The total volume of the reservoir was 200 mL. The temperature (37°C), pH (7.4), and perfusion pressure (14 mm Hg) were periodically observed. They remained stable and constant throughout the study. Liver viability was verified via the liver enzyme activities monitoring (AST and ALT). Perfusate samples were collected at 10 minutes intervals up to 60 minutes, and then 15 min intervals up to 120 minutes. After that, the samples centrifuged and the upper transparent section of the samples were isolated.

All given samples were frozen at -72°C temperature to the time of analysis.

### Bioanalytical procedures

**Figure 3:**
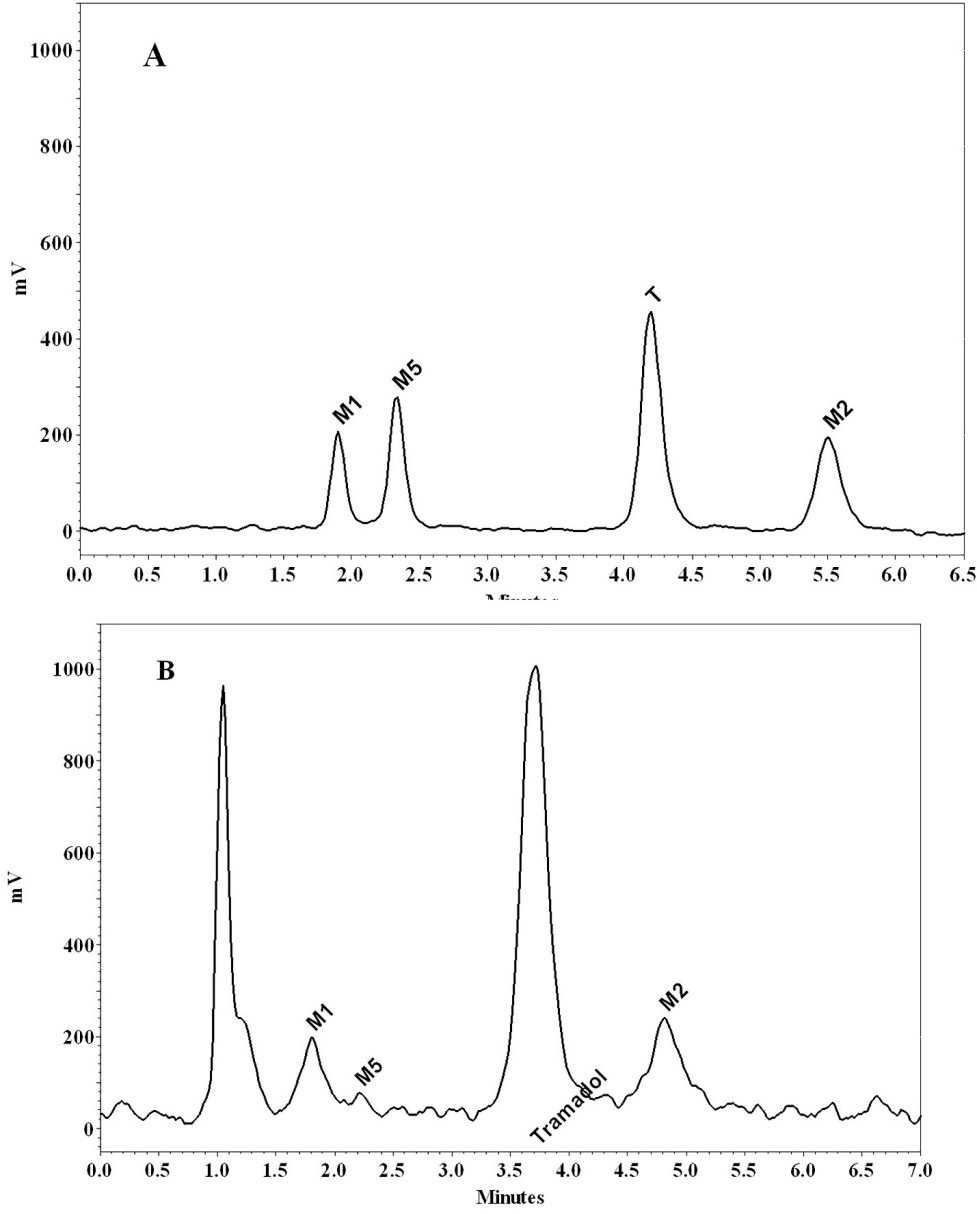
Chromatograms of tramadol (T), M1, M2 and M5 in a standard solution (200 ng/mL) of tramadol and its metabolites (A), and a perfusate sample using a 10 mg/kg intraperitoneally dose of tramadol (B)

To determine the concentration of tramadol and its metabolites in perfusate samples, a previously developed high-performance liquid chromatography (HPLC) was utilized.^28^ Briefly, all the samples were injected into a Knauer HPLC (Berlin, Germany), equipped with a low-pressure gradient HPLC pump, a fluorescence detector, a Rheodyne injector with a 100 μL loop and an online degasser. Excitation and emission wavelengths were read at 201 nm and 302 nm, respectively. To separate the analytes, a Chromolith^TM^ performance RP-18e 100 × 4.6 mm column (Merck, Darmstadt, Germany) protected by a Chromolith^TM^ guard cartridge RP-18e 5 × 4.6 mm was used. The mobile phase was a mixture of water (adjusted to pH 2.5 with orthophosphoric acid) and methanol (81:19, v/v) and was delivered with a flow rate of 2 mL/min. Data acquisition was carried out by using ChromGate software (Knauer, Berlin, Germany). The whole run time was about 6 minutes, and the retention times of M1, M5, Tramadol, and M2 were 1.8, 2.2, 3.9, and 5.2 minutes, respectively (Figure 3).

### Pharmacokinetic analysis

The calibration curve and related equation for tramadol and its metabolites were prepared by using the QC concentrations of tramadol and its metabolites. Also, the concentration of the analytes in perfusate samples was determined, at any time, using the equation mentioned before. The area under the concentration (AUC) versus time curve was calculated by the trapezoidal rule, from zero to time t. The metabolite ratio was equal to the AUC_0-t_ of each metabolite at the time of t, which was divided by the AUC_0-t_ of tramadol at the same time.

### Statistical analysis

The statistical student *t* test, with a significance level of 0.05, was used to analyze the differences between the data sets (Microsoft Office Excel 2010 software). The data are presented as mean ± SD. Microsoft Office Excel 2010 software was also used to draw the graphs.

## Results and Discussion

MDMA or ecstasy is one of the synthetic derivatives of amphetamine. It has a similar structure and function to amphetamines.^1^ According to the literature review, the global number of MDMA users in 2017 was estimated to 21.3 million of the population aged 15-64 (based on the report of UNODC).^2^ MDMA has been reported as the second drug of abuse after marijuana.^29^ Thus, MDMA consumption is an important issue around the world.

It has been reported that due to the presence of the methylenedioxy group in its structure, ecstasy irreversibly inhibits CYP2D6 (mechanism-based inhibition).^11,12^ It may also have the same effect on the other enzymes involved in its metabolism, including CYP3A4. By considering the possible simultaneous consumption of MDMA and tramadol, and also the involvement of both CYP2D6 and CYP3A4 isoenzymes in tramadol metabolism, it seemed worthwhile to investigate their interaction, focusing on enzymes involved in their metabolism. Several articles are expressing the role of MDMA in CYP2D6 inhibition. In addition, several studies considered tramadol as a CYP2D6 probe.^11,12,14,15,19,30^ However, there are a few studies particularly focusing on the effect of ecstasy on the function of CYP3A4.^21,22^ The present study, using an isolated perfused rat liver model, is the first study to investigate tramadol and MDMA interaction by focusing on both the CYP2D6 and CYP3A4 activity.

MDMA treatment groups were intraperitoneally administrated different doses of ecstasy (1, 5, and 10 mg/kg) in three subgroups (four rats in each subgroup). One hour after MDMA (treatment groups) and normal saline (control group) administration,^8^ all the animals’ livers were perfused with fresh Krebs-Henseleit buffer, containing tramadol in a concentration of 1 µg/mL.

**Figure 4:**
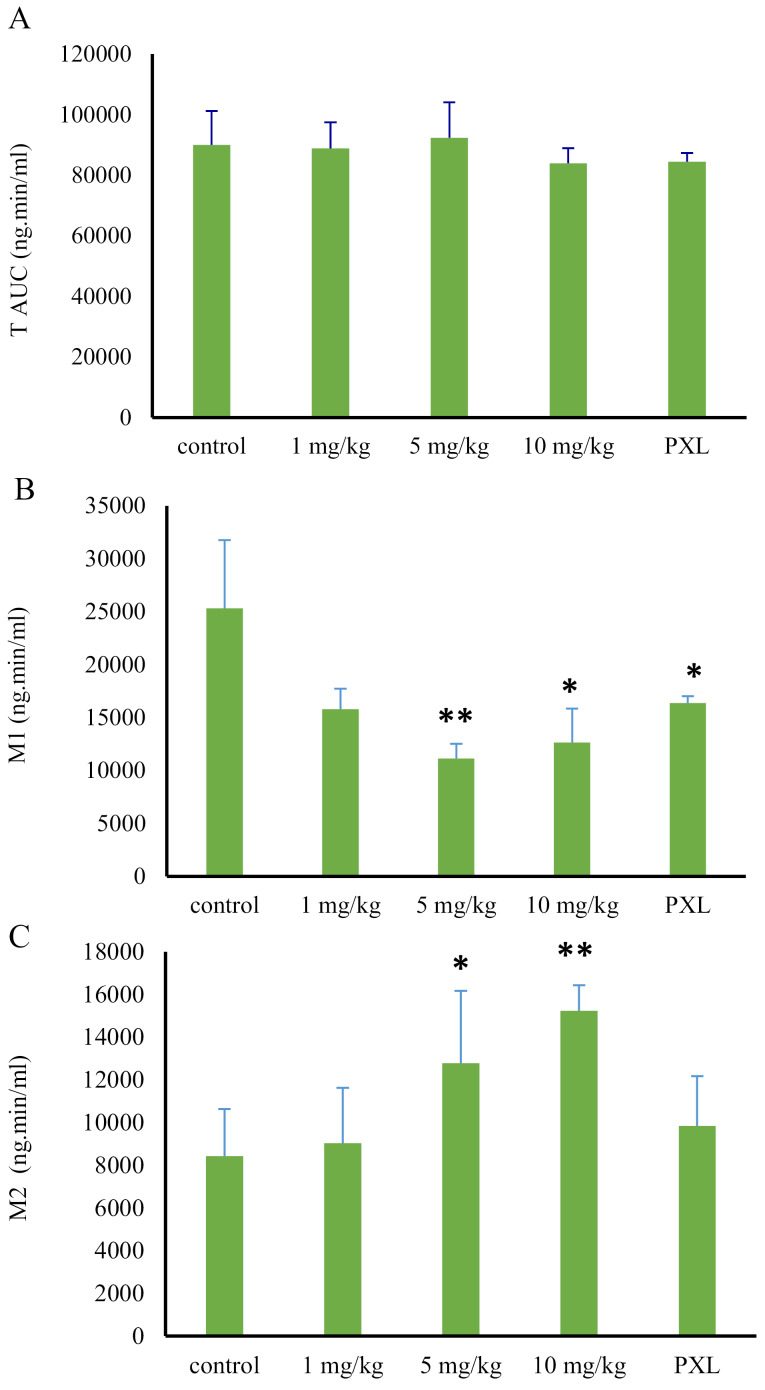
Comparison of tramadol, M1, and M2 AUCs in treatment, paroxetine, and control groups, following liver perfusion with a 1 µg/mL dose of tramadol (* and **: a significant difference of *P* > 0.05 and *P* > 0.01, respectively).

To compare the pharmacokinetic parameters, between the control and treatment groups, values of the AUC and the metabolite ratio (based on AUC of each metabolite to tramadol AUC) were used. The AUC of tramadol and its metabolites (M1 and M2), in control and treatment animals (following 1,5 and 10 mg/kg MDMA administration), was shown in Figure 4. According to the outcome, there were no significant differences among the tramadol AUCs, among the mentioned groups (*P* value > 0.05). However, the AUC_0-t_ of M1 metabolite met significant reductions following MDMA consumption (*P* value > 0.05). Animals who received 1, 5 and 10 mg/kg doses of MDMA, encountered the reductions of 38%, 56%, and 50%, respectively, in M1 concentration, compared to the control group (Figure 4, Table 1).

**Table 1:** The impact of different doses of MDMA (1-10 mg/kg) or paroxetine (0.266 mg/kg) on the AUC and metabolite ratio of M1and M2 following isolated liver perfusion of 1 µg/mL of tramadol (mean ± SD, n=4)

	**AUC**		**Metabolite ratio**	
	**M1 %** ^a^	**M2 %** ^b^	**M1 %** ^a^	**M2 %** ^b^
1 mg/kg	38*	14	29	46
5 mg/kg	56*	58*	51*	62*
10 mg/kg	50*	78*	45*	101*
PXL	40*	10	30*	33

PXL, paroxetine * Significant difference from control. ^a^ Values represent the decreased percentages compared to the control group. ^b^ Values represent the increased percentages compared to the control group.

On the other hand, AUC of M2 metabolite increased, following the MDMA administration in a dose range of 1 to 10 mg/kg. In the first group which received MDMA with a dose of 1 mg/kg, the M2 AUC increased only about 14%, compared to the control group (*P* value > 0.05). However, following the administration of larger MDMA doses (5 and 10 mg/kg), AUC showed 58% and 78% increase compared to the control group (*P* value > 0.05) (Figure 4, Table 1).

**Figure 5:**
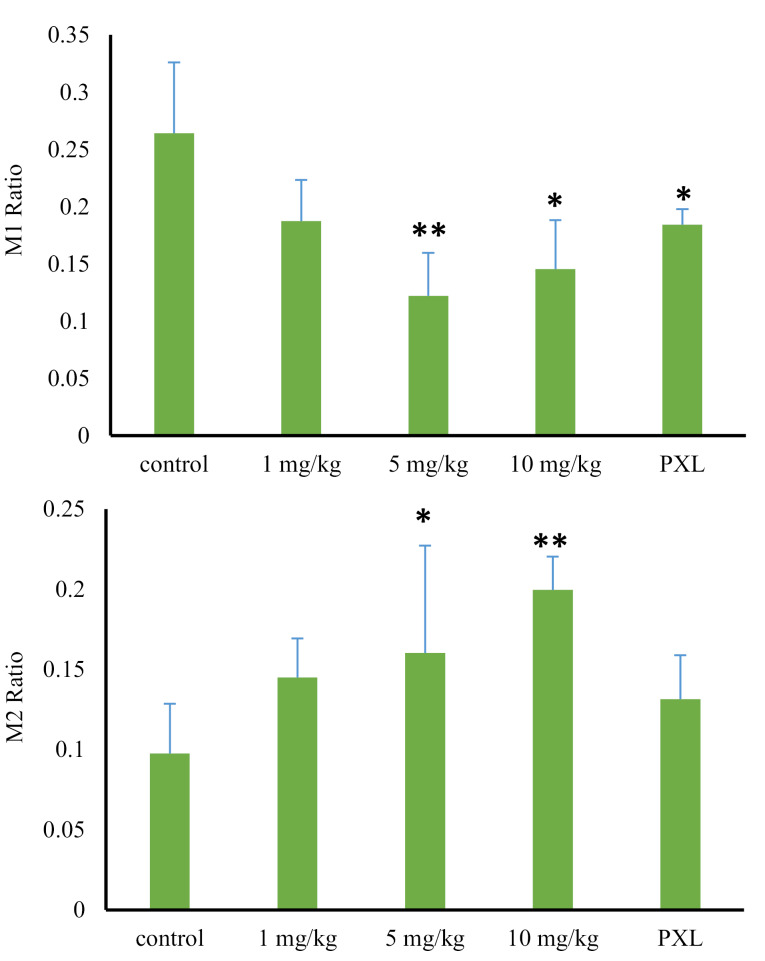
Comparison of metabolite ratios for M1 and M2 in treatment, paroxetine, and control groups, following liver perfusion with a 1 µg/mL dose of tramadol (* and **: a significant difference of *P* > 0.05 and *P* > 0.01, respectively).

Despite the 29% reduction in the 1 mg/kg group (0.19 ± 0.03) compared to the control group (0.27 ± .0.06), the metabolite ratio of M1 was not statistically decreased (*P* = 0.078). However, the metabolite ratio of M1 significantly decreased, following the administration of larger doses of the MDMA. Compared with the control group, the reductions of 51% (0.13 ± 0.03) and 45% (0.14 ± 0.03) were observed after administration of 5 mg/kg and 10 mg/kg doses of MDMA, respectively (*P* > 0.05) (Figure 5, Table 1).

According to these results, by increasing the MDMA dose, the inhibitory effect of ecstasy on CYP2D6 increased. However, the maximum inhibitory effect of MDMA was observed from a dose of 5 mg/kg (AUC of M1 and M1 metabolite ratio decreased by about 50%), and after that with increasing the dose, no more significant inhibition increase was observed. According to the amount of M1 decrease and incomplete M1 production blockage, it can be hypothesized that other important enzymatic reactions also contribute to M1 production which is not inhibited by MDMA.

On the contrary, by increasing the dose of ecstasy, M2 production was increased. Despite a 46% increase (0.151 ± 0.02) in metabolite ratio of M2 in 1 mg/kg group compared to the control group (0.103 ± 0.032), the observed increase was not statistically significant (*P* value = 0.056). However, this valueencountered a significant increase after the administration of higher MDMA doses. This increase was 62% (0.167 ± 0.06) and 101% (0.207 ± 0.02) following 5 mg/kg and 10 mg/kg MDMA administration, respectively (*P* value > 0.05) (Figure 5, Table 1). M2 production is catalyzed by CYP3A4 isoenzyme activity. However, regarding previous studies, MDMA is a potent CYP2D6 inhibitor^14^. Three hypotheses may be able to explain the observed phenomenon relating to the increase of M2 production; the metabolism shifts, lack of M2 consumption, and induction of CYP3A4 isoenzyme by the ortho-quinone groups made by MDMA metabolism. In the first hypothesis, when the CYP2D6 activity is inhibited by MDMA, the M1 production decreases. Therefore, and in order to compensate for the decrease, a shift in metabolism to M2 production may happen. In the second hypothesis, due to the inhibition of CYP2D6 and its role in the conversion of M2 to M5, the M5 metabolite production pathway was blocked. Thus, the M2 did not consume, and its level remained high. In the third hypothesis, CYP3A4 induced by the intermediate ortho-quinone groups made by MDMA metabolism.

Further investigation was needed in order to accept or reject each of these hypotheses. For this purpose, another control group (positive control) was added to the present study, where the CYP2D6 was inhibited by paroxetine as a potent, selective CYP2D6 inhibitor. However, the CYP3A4 activity was remained unchanged. Since there was no animal study investigating the paroxetine effects on CYP2D6 in rats, the applied dose in the present study was extracted from human studies. In most studies, 20 mg dose has been given to the patients, daily, (weight of 75 kg) for three consecutive days. We applied the dose of 0.266 mg/kg, which is similar to the human dose.^27^

Compared to the control group, the AUC of tramadol in the paroxetine group did not significantly change. However, M1 AUC value faced a significant reduction of 40% (16378.2 ± 630.2) when being compared to the control group (25309.8 ± 6436.6) (*P* value = 0.03). The amount of decrease in M1 production was similar to the results of ecstasy administration in a dose of 1 mg/kg, demonstrating the complete inhibition of cytochrome CYP2D6 by a minimum dose of ecstasy. Therefore, the involvement of the other liver isoenzymes in M1 production is proved. Moreover, the AUC of M2 increased by 10%, which was not significantly different from that of the control group (*P* value = 0.4) (Figure 4). The metabolite ratio of M1 reduced by 30% (0.189 ± 0.01) in comparison to the control group (0.27 ± . 0.06) (*P* value = 0.047). On the contrary, the metabolite ratio of M2 raised by 33% (0.137 ± 0.02) compared to that of control group (0.103 ± 0.03) (P-value = 0.16) showing an insignificant increase (Figure 5, Table 1).

As stated before, M1 and M2 are N- and O- demethylated by CYP3A4 and CYP2D6, respectively, to produce M5 as a secondary metabolite. Due to the lack of M5 production in all the treatment groups, it can probably be concluded that M5 is mainly produced from O- demethylation of, M2 and its production was blocked by CYP2D6. Full inhibition resulted from MDMA administration. Inhibition of the mentioned pathway would be involved in the elevation of the M2 level. Although the amount of M2 in all the treatment groups was significantly higher than that of the control group, the observed elevation was not significantly different between those groups of animals receiving MDMA doses of 1 and 5 mg/kg. Following the comparison among the M2 production in the 1 and 10 mg/kg ecstasy, and animals receiving paroxetine (*P* value > 0.05), the involvement of another mechanism would be postulated for explaining the increase in the M2 following a 10 mg/kg dose of MDMA. The results demonstrated that the complete inhibition of cytochrome CYP2D6 at a dose of 1 mg/kg group of ecstasy, and M2 metabolite production was not significantly increased in the paroxetine group. Consequently, it seems that the elevation of M2 production would not be a result of the metabolism shift.

## Conclusion

According to the high amount of CYP3A4 and its functional role in drug metabolism, at least in common doses of ecstasy, it is not inhibited. On the other hand, the induction of CYP3A4 cannot be completely ruled out. However, it should be considered that many other isoenzymes involved in the metabolism of tramadol and ecstasy, may affect them and result in an increased production of M2.

## Ethical Issues

All procedures performed in studies, involving animals, were following the ethical standards of the Animal Ethics Committee and Institutional Review Board of Pharmaceutical Research Centre of Tehran University of Medical Science.

## Conflict of Interest

The authors declare that they have no conflict of interest.

## Acknowledgments

This work was part of a Pharm.D thesis supported by Tehran University of Medical Sciences. The experimental procedures were done in the Faculty of Pharmacy, Tehran University of Medical Sciences.
